# The Story of the Insane from Year to Year

**Published:** 1893-12-30

**Authors:** 


### THE STORY OF THE INSANE FROM YEAR TO YEAR.

### CHESTER COUNTY ASYLUM AT UPTON.

An increase of 11 cases has to be noted; and the asylum had in residence at the close of 1892 613 patients. One hundred and seventy cases were admitted, 74 were discharged recovered, and 70 died. Calculated in the usual way these numbers give a recovery rate of 43·5, and a death-rate of 12·9, both being above the average in asylums. Turning to Table X., which gives the causes of insanity in the year's admissions, we find that 24 were driven mad by drink, and 16 by hereditary influence, while "previous attack" is the assigned cause in 34. Ten deaths were caused by phthisis, and this disease existed as a complication in two other cases. Post-mortem examinations were made in 64 cases, and Dr. Davidson tells us he was in one of these threatened with legal proceedings by the relatives of the deceased. We have ere now had to comment on the wholesale making of post-mortem examinations in our county asylums, and we think Dr. Davidson is very fortunate to have escaped with only a threat of legal proceedings. That post-mortem examinations ought to be made in cases where there is a doubt as to the cause of death, and in cases where something may be learned, is beyond controversy; but many, perhaps the majority, of asylum post-mortems come under neither of these heads, and are, therefore, unjustifiable. The asylum is full, and it has been decided to build an annexe for 400 patients. The committee testify "to the admirable and economic management of Dr. Davidson," and the Commissioners say they "can report favourably of the general condition of the asylum." The weekly cost of maintenance is barely 6s. 10½d. per patient, a sum which we regard as decidedly too low.

### SUSSEX COUNTY ASYLUM.

The numbers increased during the year 1892 from 792 to 857, and this does not include 140 boarded out at the Northampton Asylum and 15 at Oxford. There were 283 admissions, 50 recoveries, 36 discharged "relieved," and 123 deaths. These numbers show that the recoveries stand at 18·8 per cent. on the admissions, and the deaths at 15·1 on the average number resident. The former is below the average in county asylums, and the latter above it. Fifty of the admissions were owing to hereditary influence, and 17 to drink, but as 85 are put down as "unknown" there can be no doubt that the heredity and drink curses should stand higher. Turning to Table V., which details the causes of death, we find that 52 were caused by brain diseases and 17 to consumption. Dr. Saunders points out the "solemn farce" of recertifying 525 chronic lunatics, and he says, "The Legislature are very slow to recognise the weakness, inconsistency, and contradictions of the Lunacy Act of 1890." This is too true. No one seems inclined to take the initiative and do his best to wipe the miserable Act off the statute book. It will be seen that the Sussex Asylum is more than full. The partnership between the east and west divisions of the county terminates at the end of 1893, and Dr. Saunders looks forward to the time when he will be rid of the Brighton patients. Twelve of the attendants and nurses have passed the examination instituted by the Medico-Psychological Society. The certificates were framed, and presented to the candidates by the chairman. Three nurses and one attendant received pensions during the year. The weekly rate of maintenance is 9s. 2½d.

### KENT COUNTY ASYLUM AT BARMING HEATH.

On the last day of 1892 there were in residence here the high number of 1,513 patients. The admissions were 388, the recoveries 182, and the deaths 171. The percentages of recoveries stands at 51·4, and that of the deaths at 11·1. Only 18 of the admissions are stated to be caused by drink, and only 22 are supposed to be owing to hereditary taint, but it is absolutely certain that investigation on these points would double or treble these causes. Indeed, we find that 125 are put down as "unknown," so that the table is practically useless. Fifty-four of the deaths were due to some kind of brain disease, 37 to phthisis, 18 to pneumonia, 6 to chronic bronchitis, and 3 to pleurisy. Dr. Davies mourns the fact that he was able to make only 126 post-mortem examinations out of 171 deaths, but we thinks he made a great many more than was in all probability necessary, and as for his suggestion that the law should require such examinations in every case of death, we should regard this as an unjustifiable trampling on the feelings of the relatives of the insane. The visitors report that the state and condition of the asylum are good, and that it is sufficient to provide the necessary accommodation for all the patients now confined therein; whereas the Commissioners in Lunacy say that no Kent patient has yet been refused admission, but refusals have only been avoided, we think, by overcrowding. Of course, many foreign patients might have been cleared out between the date of the Commissioners' report (April 23rd, 1892) and that of the visitors (February 8th, 1893). The Commissioners suggest that each room should be measured and its cubic contents known. This is an admirable suggestion. It would be well if it were carried out in every asylum in England, and the space printed on the doors of the rooms. The Commissioners further think the supply of newspapers might be increased. The weekly rate is 8s. 11¾d.

